# Endothelin-1 is increased in the plasma of patients hospitalised with Covid-19

**DOI:** 10.1016/j.yjmcc.2022.03.007

**Published:** 2022-06

**Authors:** George R. Abraham, Rhoda E. Kuc, Magnus Althage, Peter J. Greasley, Philip Ambery, Janet J. Maguire, Ian B. Wilkinson, Stephen P. Hoole, Joseph Cheriyan, Anthony P. Davenport

**Affiliations:** a Royal Papworth Hospital NHS Foundation Trust, Cambridge Biomedical Campus, Cambridge, UK; b Division of Experimental Medicine and Immunotherapeutics, University of Cambridge, Addenbrooke's Hospital, Cambridge, UK; c Late-stage Development, Cardiovascular, Renal and Metabolism (CVRM), BioPharmaceuticals R&D, AstraZeneca, Gothenburg, Sweden; d Clinical Pharmacology Department and Cardiovascular Office, Cambridge Clinical Trials Unit, Cambridge University Hospitals NHS Foundation Trust, UK

**Keywords:** Endothelin-1, Covid-19, Endotheliitis, Endothelin receptor antagonists

## Abstract

Virus induced endothelial dysregulation is a well-recognised feature of severe Covid-19 infection. Endothelin-1 (ET-1) is the most highly expressed peptide in endothelial cells and a potent vasoconstrictor, thus representing a potential therapeutic target.

ET-1 plasma levels were measured in a cohort of 194 Covid-19 patients stratified according to the clinical severity of their illness. Hospitalised patients, including those who died and those developing acute myocardial or kidney injury, had significantly elevated ET-1 plasma levels during the acute phase of infection.

The results support the hypothesis that endothelin receptor antagonists may provide clinical benefit for certain Covid-19 patients.

## Highlights

• Dysregulation of endothelial function is a feature of severe Covid-19 infection.
• The endothelial peptide, ET-1 is a potential therapeutic target.
• Higher circulating ET-1 levels are associated with more severe infection.
• Endothelin receptor antagonists may be of clinical benefit in Covid-19.

## Graphical abstract

**Figure:**
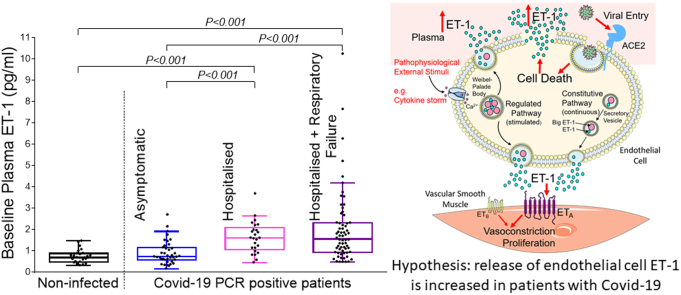

## 1 Introduction

The Coronavirus disease 2019 (Covid-19) pandemic continues to place a devastating strain on healthcare services worldwide and there remains an ongoing requirement for new therapies [1].

SARS-CoV-2, the causative agent of Covid-19, infects host cells by exploiting ACE2, a surface receptor expressed by epithelial cells and the vascular endothelium – the layer of cells lining all blood vessels [1]. There is emerging consensus that in progressive severe Covid-19 disease, virus-induced endothelial damage may result in a syndrome of excessive vasoconstriction, inflammation and thrombosis [2,3]. Viral inclusions and lymphocytic infiltration of apoptotic endothelial cells have been identified from histological examination of tissues obtained from Covid-19 patients with acute lung [4,5], kidney [5] and myocardial injury [5,6]. Additionally, multiple plasma markers for endothelial injury are elevated in hospitalised Covid-19 patients on admission [7,8].

Endothelin-1 (ET-1), being the most highly expressed peptide in endothelial cells and potent vasoconstrictor of human blood vessels [9,10], represents a potential therapeutic target. The benefit of endothelin receptor antagonists is already well established in pulmonary arterial hypertension [11] hence these medications may be suitable for accelerated regulatory approval. ET-1 is released from endothelial cells via a continuous constitutive pathway and supplemented by ET-1 release from Weibel-Palade bodies (the unique storage granules of endothelial cells) in response to extra-cellular stimuli including inflammatory cytokines [10].

Willems et al. have recently reported elevated ET-1 levels in patients 3 months post Covid-19 infection [12]; however the association of ET-1 with clinical outcomes likely to impact the provision of healthcare resources, such as hospitalisation, has not been investigated. In the present study, we tested the hypothesis that elevated levels of plasma ET-1 in the acute phase of Covid-19 infection would be associated with more severe disease.

## 2 Methods

Plasma samples and clinical variables were obtained with ethical approval (REC:17/EE/0025) and informed consent from participants enrolled in the Cambridge NIHR Covid-19 Biobank project. Patients with positive SARS-Cov-2 PCR tests were divided into three groups based on the clinical severity of their illness: A, asymptomatic or mild symptoms not requiring hospitalisation; B, symptoms requiring hospitalisation but with no requirement for supplemental oxygen therapy at any stage; C, hospitalised with symptoms requiring one, or a combination of supplemental oxygen therapy, non-invasive or invasive ventilation. Patient demographics and clinical codes for underlying comorbidities (hypertension, ischemic heart disease, diabetes, congestive cardiac failure and chronic kidney disease) were retrospectively obtained from the electronic medical record as was the occurrence during the index admission of the following clinical endpoints: acute kidney injury (defined according to the Kidney Disease Improving Global Outcomes guidelines (2012): an acute rise in serum creatinine ≥26.5 μmol/l from baseline), acute myocardial injury (defined according to the Fourth Universal Definition of Myocardial Infarction (2018): an acute rise in cardiac troponin level with at least one value >99th percentile upper reference limit) and inpatient mortality. Venous blood samples were collected (where possible after the time of enrolment) from patients at 0, 28 and 90 days after admission to hospital (or from the time of PCR test for Group A). Samples were also collected at baseline for a control group comprising PCR negative hospital staff. ET-1 was measured in duplicate using an established sandwich ELISA (R&D Systems, U.S.A).

Statistical analysis was performed using SPSS version 27 (IBM Corp., USA) for Windows. Normality of continuous variable distributions was tested by one-sample Kolmogorov-Smirnov test. Non-normally distributed continuous variables are presented as Median (inter-quartile range [Q1 - Q3]). Pairwise comparison of ET-1 levels between patient categories A-C and controls, between different time-points for categories A-C, and between subgroups defined by clinical endpoints was undertaken using the Independent Samples Kruskal-Wallis Test. To assess for potential confounding effects when comparing ET-1 levels between patient categories A-C and controls as well as between all Covid-19 infected patients with controls, univariate analysis of co-variance was performed for each of: age, gender, ethnicity, hypertension, ischemic heart disease, diabetes, congestive cardiac failure and chronic kidney disease. To assess the association of baseline ET-1 with hospitalisation amongst infected patients, a binary logistic regression model was calculated adjusted for recognised confounders of baseline ET-1: hypertension, ischemic heart disease, diabetes, congestive cardiac failure and chronic kidney disease.

## 3 Results

**Fig. 1:**
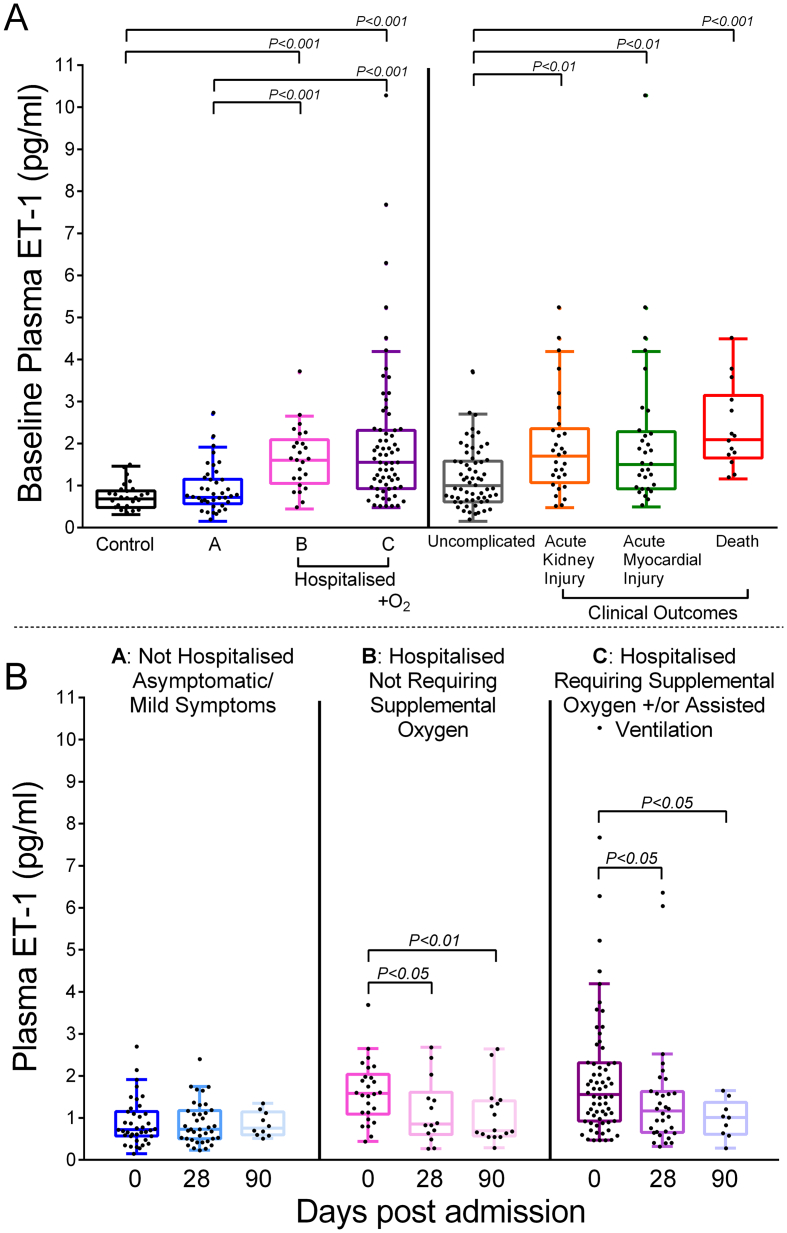
A, Comparison of ET-1 at baseline across categories: Controls refer to non-infected volunteers (*n* = 26); group A (*n* = 51): non-hospitalised Covid-19 patients; groups B (*n* = 39) and C (*n* = 78): hospitalised Covid-19 patients; uncomplicated (*n* = 62): Covid-19 infected patients after excluding dying patients (*n* = 14), patients requiring supplemental oxygen or assisted ventilation (n = 78) or developing acute kidney injury (*n* = 29) or acute myocardial injury (*n* = 31). ET-1 concentrations represent mean concentration following measurement in duplicate using ELISA (R&D Systems, U.S.A). B, Comparison of ET-1 levels in categories A-C at day 28 and 90 after admission compared to day 0.

All data that support the findings of this study are available from the corresponding author upon reasonable request. Plasma samples were obtained from 194 patients. Samples from 157 patients were available at baseline, 84 patients at day 28 and 35 patients at day 90. Reasons for drop-out during follow-up included death, persisting disability and repatriation outside our locality, having been initially referred to our centre for specialist care. Plasma samples were sufficient for analysis in all cases. At baseline, ET-1 levels (pg/ml) were significantly elevated in the patients requiring hospitalisation (Group B: 1.59 [1.13–1.98], and C: 1.65 [1.02–2.32]) compared with both controls (0.68 [0.47–0.87], *p* ≤0.001) and patients with asymptomatic or mild infection (Group A: 0.72 [0.57–1.10], p ≤0.001): Fig. 1A. Mean age ± SD was 41.1 ± 16.4 in controls, 35.8 ± 12.6 in group A, 58.8 ± 17.1 in group B and 62.1 ± 14.3 in Group C. The proportion of females was 61.5% in controls, 82.4% in group A, 38.5% in Group B and 32.1% in Group C. The frequency of underlying comorbidities was higher in Groups B and C compared with Group A and controls (Table 1). Despite heterogeneity in clinical and demographic characteristics, differences in baseline ET-1 between infected versus non-infected patients and all patient categories remained significant (*p* < 0.05) in corrected models for age, gender, ethnicity, hypertension, ischemic heart disease, diabetes, congestive cardiac failure and chronic kidney disease using between-subjects effect analysis of co-variance (Table 2*)*. Amongst the infected patients, baseline ET-1 was a significant independent predictor of hospitalisation in a multivariate binary logistic regression model including hypertension, ischemic heart disease, diabetes, congestive cardiac failure and chronic kidney disease (odds ratio [95%CI]: 4.5 [1.8–11.2], *p* = 0.001 - *appendix*).

**Table 1:** ET-1, patient demographics and clinical endpoints.

Time-point	controls	A	B	C
	*n* = 26	*n* = 51	*n* = 39	*n* = 78
ET-1 day 0	0.68 (0.47–0.87)	0.72 (0.57–1.10)	1.59 (1.13–1.98)^⁎⁎⁎^	1.65 (1.02–2.32)^⁎⁎⁎^
*n* = 157				
ET-1 day 28	–	0.73 (0.50–1.18)	0.86 (0.60–1.61)^†^	1.17 (0.66–1.62)^†^
*n* = 84				
ET-1 day 90	–	0.76 (0.60–1.12)	0.69 (0.59–1.38)^†^	1.01 (0.64–1.21)^†^
*n* = 35				

Demographics				
Age (Mean ± SD)	41.1 ± 16.4	35.8 ± 12.6	58.8 ± 17.1	62.1 ± 14.3
Female	16 (62)	42 (82)	15 (39)	25 (32)
White ethnicity	21 (80)	40 (78)	32 (84)	64 (82)
HTN	3 (12)	2 (4)	16 (44)	36 (46)
IHD	0 (0)	0 (0)	4 (11)	14 (18)
DM	1 (4)	0 (0)	9 (25)	31 (40)
CCF	0 (0)	0 (0)	2 (6)	10 (13)
CKD	0 (0)	0 (0)	7 (19)	15 (20)

Clinical Endpoints				
AKI	–	–	3 (8)	30 (38)
AMI	–	–	4 (11)	34 (45)
Death	–	–	1 (3)	13 (17)
Admission duration (days)	–	–	6.7 ± 11.6	31.8 ± 3.8
cTn (ng/L)	–	–	5.5 (0.0–13.9)	16.2 (5.4–59.2)
NTproBNP (pg/ml)	–	–	113 (35–258)	313 (123–1315)

Table shows: *Top*: ET-1 (pg/ml) at all time-points (median [IQR]). ^⁎⁎⁎^ indicates significant difference (p ≤0.001) when comparing indicated patient group and control; ^†^ indicates significant difference (p ≤0.05) when comparing ET-1 at the indicated time-point with the corresponding baseline ET-1 level. *Middle*: Demographics and underlying comorbidities within patient categories, figures are n (% of total). HTN indicates hypertension, IHD: ischemic heart disease, DM: diabetes mellitus, CCF: congestive cardiac failure, CKD: chronic kidney disease. *Bottom*: Clinical endpoints recorded for hospitalised patients, figures are n (% of total). AKI indicates acute kidney injury, AMI: acute myocardial injury. cTn refers to peak cardiac specific Troponin levels (median [IQR]); NTproBNP: peak N-terminal pro-B-type natriuretic peptide (median [IQR]); Admission duration: duration of index Covid-19 related admission measured in total days (mean ± SD).

**Table 2:** Differences in baseline ET-1 adjusted for confounding using univariate analyses of co-variance.

Null hypothesis: Baseline ET-1 is not significantly different:	between patient categories (controls, A, B and C)		between infected (A, B and C) and non-infected patients (controls)	
Covariate Variables	Test statistic	*p* value after univariate adjustment	Test statistic	p value after univariate adjustment
Age	2.91	0.04	6.02	0.04
Gender	13.4	<0.001	17.1	<0.01
Ethnicity	16.0	<0.001	17.5	<0.01
HTN	7.3	<0.001	12.1	<0.01
IHD	8.9	<0.001	13.7	<0.01
DM	7.5	<0.001	12.0	<0.01
CCF	7.8	<0.001	11.9	<0.01
CKD	9.0	<0.001	13.7	<0.01

Differences in baseline ET-1 remained significant after adjustment for confounding variables. Test statistic: difference in mean square ET-1 at time 0 after adjustment for covariate variables; p value after univariate adjustment refers to probability that differences in baseline ET-1 are not significantly different between patient categories (controls, A, B and C) and between infected and non-infected patients after adjustment for differences in covariate variables.

Baseline ET-1 levels (pg/ml) were also significantly elevated in subgroups of patients that died (2.09 [1.66–3.15]), developed acute kidney (1.70 [1.07–2.36]) or myocardial injury (1.50 [0.92–2.28]) compared with patients with an uncomplicated infection (1.00 [0.61–1.57], *p* ≤0.01) for whom these endpoints were not recorded (Fig. 1A).

Amongst surviving hospitalised patients, ET-1 levels (pg/ml) decreased monotonically when measured at 28 days (Group B: 0.86 [0.60–1.61] and Group C: 1.17 [0.66–1.62] versus baseline, *p* ≤0.05) and 90 days (Group B: 0.69 [0.59–1.38] and Group C: 1.01 [0.64–1.21] versus baseline, p ≤0.05): Fig. 1B.

## 4 Discussion

**Fig. 2:**
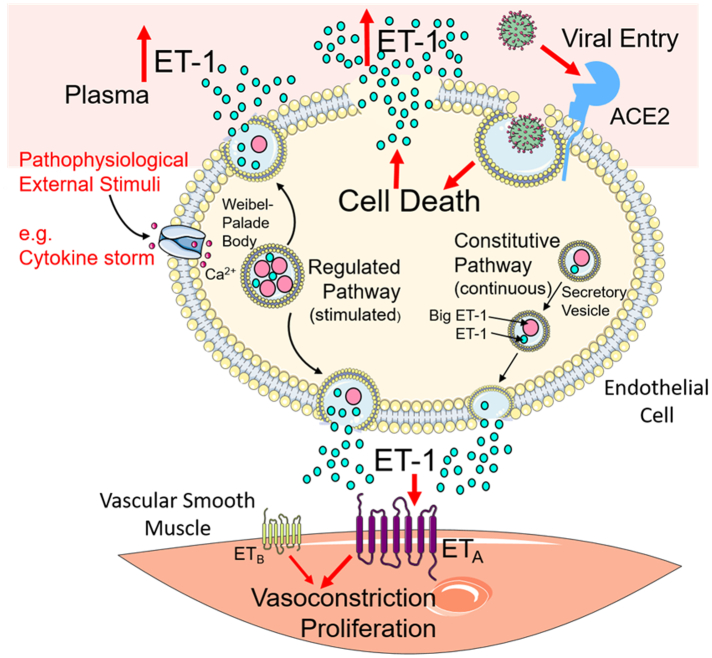
Hypothesis for mechanism of increased plasma ET-1 levels in severe Covid-19 infection. Covid-19 infection may up-regulate circulating cytokines to stimulate the regulated ET-1 secretory pathway. Additionally, viral entry into endothelial cells induces cell damage and release of stored ET-1 into the circulation. (Illustration generated using Servier Medical Art (smart.servier.com) used under Creative Commons License CC BY 3.0).

Our results demonstrate a dynamic virus induced increase in the level of the endothelial peptide, ET-1 during the acute phase of Covid-19 infection, which is associated with the clinical severity of disease. We hypothesise that elevation of ET-1 plasma levels could result from inflammatory cytokine mediated up-regulation of the stimulated Weibel-Palade body ET-1 secretory pathway. Von-Willebrand Factor is the principal stored component of Weibel-Palade bodies [13] and has been shown to be similarly up-regulated in the acute phase of Covid-19 infection [8]. Alternatively or additionally, viral induced cell death may result in the release of stored ET-1 into the circulation (Fig. 2).

Accumulating evidence suggests endothelial dysregulation is a key mechanism for disease progression following Covid-19 infection. Post-mortem histology from Covid-19 patients with multi-organ failure has identified pathological endotheliitis in multiple vascular beds [[4], [5], [6]] while elevation of various biomarkers of endothelial damage in patients with severe Covid-19 infection has also been demonstrated [7,8].

Our results are replicated by Willems et al. who reported an elevation in ET-1 levels for a subset of 36 Covid-19 infected patients during the acute illness compared with a historic non-infected control group [12]. Gregoriano et al. had earlier reported on the lack of prognostic utility of C-terminal proendothelin-1 (proET-1) to predict mortality in Covid-19 [14]. Compared with ET-1 measured in the present study, proET-1 is metabolised differently and is an inactive peptide that does not cause vasoconstriction therefore significant correlation between the two biomarkers is unlikely in vivo.

Higher plasma levels of ET-1 are a recognised feature of pulmonary arterial hypertension (PAH) and increased expression of ET-1 in pulmonary endothelial cells has been shown to correlate with increased pulmonary vascular resistance in this condition [11]. The magnitude of the ET-1 elevation we have observed in hospitalised Covid-19 patients was similar to that reported in the early studies comparing ET-1 levels in PAH patients with normal subjects [15] [16]. Multiple large clinical trials have now established the prognostic benefit of ET receptor antagonists in idiopathic and connective tissue disease associated PAH [11]. Outside of PAH, increased plasma levels of ET-1 are also associated with increased coronary [17] and systemic vasoconstriction [18] in coronary microvascular dysfunction, and endothelial dysfunction in vasospastic angina [19] highlighting that ET-1 dysregulation affects disparate vascular territories with adverse consequences. In our Covid-19 cohort, the highest plasma ET-1 levels were seen in patients who died in hospital. Levels were also high in patients whose infection was complicated by respiratory failure (indicated by a requirement for supplemental oxygen or assisted ventilation), acute kidney or myocardial injury. This generates the hypothesis that increased circulating ET-1 and ET_A_ receptor activation may lead to increased vasoconstriction in different vascular beds contributing to progressive Covid-19 disease.

### 4.1 Limitations

This was a retrospective observational study with all patients enrolled during the very challenging first wave of the pandemic, when no specific therapies for Covid-19 were known and prior to the availability of vaccines. Further exploration of the pathophysiological changes resulting from elevated ET-1 levels in critically unwell patients was not feasible in this period. Our study was underpowered to detect significant associations of ET-1 independent from confounding variables for rarer clinical endpoints such as mortality hence this data should be considered hypothesis generating only.

## 5 Conclusion

Our results demonstrate that elevated ET-1 in the acute phase of Covid-19 infection is a clinical feature unique to severe disease, supporting the hypothesis [8] that endothelin receptor antagonists, used to treat pulmonary arterial hypertension, may be beneficial in a subset of Covid-19 patients.

## Appendix A Supplementary data

Supplementary material Image 1

## References

[1] Alexander S.P.H., Armstrong J.F., Davenport A.P. (2020). A rational roadmap for SARS-CoV-2/COVID-19 pharmacotherapeutic research and development: IUPHAR review 29. Br. J. Pharmacol..

[2] Calabretta E., Moraleda J.M., Iacobelli M. (2021). COVID-19-induced endotheliitis: emerging evidence and possible therapeutic strategies. Br. J. Haematol..

[3] Fisk M., Althage M., Moosmang S. (2021). Endothelin antagonism and sodium glucose co-transporter 2 inhibition. A potential combination therapeutic strategy for COVID-19. Pulm. Pharmacol. Ther..

[4] Ackermann M., Verleden S.E., Kuehnel M. (2020). Pulmonary vascular Endothelialitis, thrombosis, and angiogenesis in Covid-19. N. Engl. J. Med..

[5] Varga Z., Flammer A.J., Steiger P. (2020). Endothelial cell infection and endotheliitis in COVID-19. Lancet..

[6] Fox S.E., Falgout L., Vander Heide R.S. (2021). COVID-19 myocarditis: quantitative analysis of the inflammatory infiltrate and a proposed mechanism. Cardiovasc. Pathol..

[7] Thwaites R.S., Sanchez Sevilla Uruchurtu A., Siggins M.K. (2021). Inflammatory profiles across the spectrum of disease reveal a distinct role for GM-CSF in severe COVID-19. Sci Immunol..

[8] Goshua G., Pine A.B., Meizlish M.L. (2020). Endotheliopathy in COVID-19-associated coagulopathy: evidence from a single-Centre, cross-sectional study. Lancet Haematol..

[9] Davenport A.P., Hyndman K.A., Dhaun N. (2016). Endothelin. Pharmacol. Rev..

[10] Russell F.D., Davenport A.P. (1999). Secretory pathways in endothelin synthesis. Br. J. Pharmacol..

[11] Chester A.H., Yacoub M.H. (2014). The role of endothelin-1 in pulmonary arterial hypertension. Glob Cardiol Sci Pract..

[12] Willems L.H., Nagy M., Ten Cate H. (2022). Sustained inflammation, coagulation activation and elevated endothelin-1 levels without macrovascular dysfunction at 3 months after COVID-19. Thromb. Res..

[13] Rondaij M.G., Bierings R., Kragt A., van Mourik J.A., Voorberg J. (2006). Dynamics and plasticity of Weibel-Palade bodies in endothelial cells. Arterioscler. Thromb. Vasc. Biol..

[14] Gregoriano C., Damm D., Kutz A. (2021). Association of endothelial activation assessed through endothelin-I precursor peptide measurement with mortality in COVID-19 patients: an observational analysis. Respir. Res..

[15] Stewart D.J., Levy R.D., Cernacek P., Langleben D. (1991). Increased plasma endothelin-1 in pulmonary hypertension: marker or mediator of disease?. Ann. Intern. Med..

[16] Hiramoto Y., Shioyama W., Kuroda T. (2007). Effect of bosentan on plasma endothelin-1 concentration in patients with pulmonary arterial hypertension. Circ. J..

[17] Cox I.D., Bøtker H.E., Bagger J.P., Sonne H.S., Kristensen B.O., Kaski J.C. (1999). Elevated endothelin concentrations are associated with reduced coronary vasomotor responses in patients with chest pain and normal coronary arteriograms. J. Am. Coll. Cardiol..

[18] Ford T.J., Corcoran D., Padmanabhan S. (2020). Genetic dysregulation of endothelin-1 is implicated in coronary microvascular dysfunction. Eur. Heart J..

[19] Lerman A., Holmes D.R., Bell M.R., Garratt K.N., Nishimura R.A., Burnett J.C. (1995). Endothelin in coronary endothelial dysfunction and early atherosclerosis in humans. Circulation..

