# Supplementary material for: Endothelin-1 is increased in the plasma of patients hospitalised with Covid-19
Source: J Mol Cell Cardiol. 2022 Jun;167:92–6. doi: 10.1016/j.yjmcc.2022.03.007 (PMC8941861; doi:10.1016/j.yjmcc.2022.03.007)
Supplement: Supplementary file 1 — Supplementary material [file mmc1.docx]

**Appendix A: Supplementary Data**

All data that support the findings of this study are available from the corresponding author upon request.

**Supplementary information on statistical analysis**

All statistical analysis was performed using SPSS version 27 (IBM Corp., USA) for Windows. Normality of continuous variable distributions was tested by one-sample Kolmogorov-Smirnov test. Non-normally distributed continuous variables are presented as Median (inter-quartile range [Q1 - Q3]). Due to the skewed distribution of ET-1, no potential or extreme outliers have been excluded from analysis. Outliers were noted to mostly occur in patient category C (hospitalised and requiring supplemental oxygen/assisted ventilation) or amongst subgroups with clinical complications of Covid-19 infection therefore we have assumed these values reflect true biological variability in the study population. Comparison of ET-1 between patient categories was performed using the Independent Samples Kruskal-Wallis Test and also between time-points for each patient category. In the latter case, an independent samples non-parametric test was chosen to increase power given the low number of paired corresponding samples available due to patient drop-out at 28 and 90 days. Reasons for loss of patients to follow up were in many cases unavoidable including patient death, persisting disability and patients being repatriated outside our local area having been referred to our centre for specialist tertiary care during their initial illness.

No demographic or clinical endpoint data was available for 3/39 patients in category B otherwise clinical variables were available for all other enrolled patients.

**Binary Logistic Regression**

Binary Logistic Regression was used to calculate a model predicting hospitalisation for Covid-19 infected patients in our cohort using baseline ET-1, hypertension, diabetes, ischemic heart disease, congestive cardiac failure and chronic kidney disease (step 1).

| **Omnibus Tests of Model Coefficients** | | | | |
| --- | --- | --- | --- | --- |
|  | | Chi-square | df | Sig. |
| Step 1 | Step | 69.610 | 6 | p=<.0001 |
|  | Block | 69.610 | 6 | p=<.0001 |
|  | Model | 69.610 | 6 | p=<0.001 |

Chi-Square Test indicates the adjusted model incorporating all 6 covariates was significantly better than the unadjusted logistic regression model.

| **Classification Table** | | | | | |
| --- | --- | --- | --- | --- | --- |
|  | Observed | | Predicted | | |
|  |  |  | hospitalised | | Percentage Correct |
|  |  |  | not hospitalised | hospitalised |  |
| Step 1 | hospitalised | not hospitalised | 30 | 9 | 76.9 |
|  |  | hospitalised | 15 | 76 | 83.5 |
|  | Overall Percentage | |  |  | 81.5 |

Classification Table indicates the model predicted patients requiring hospitalisation 83.5% of the time and not requiring hospitalisation correctly 76.9% of the time.

| **Variables in the Equation** | | | | | | |
| --- | --- | --- | --- | --- | --- | --- |
|  | | Sig. | Exp(B) | 95% C.I.for EXP(B) | |  |
|  |  |  |  | Lower | Upper |  |
| Step 1^a^ | Baseline ET-1 concentration | .001 | 4.502 | 1.814 | 11.172 |  |
|  | hypertension | .997 | 572782954.886 | .000 | . |  |
|  | diabetes | .998 | 201570462.410 | .000 | . |  |
|  | ischemic heart disease | .998 | 89282406.212 | .000 | . |  |
|  | congestive cardiac failure | 1.000 | .002 | .000 | . |  |
|  | chronic kidney disease | 1.000 | 2.490 | .000 | . |  |
|  | Constant | .002 | .165 |  |  |  |
| a. Variable(s) entered on step 1: Baseline ET-1 concentration, hypertension, diabetes, ischemic heart disease, congestive cardiac failure,  chronic kidney disease. Exp (B) indicates odds ratio of hospitalisation vs not hospitalised for each covariate. | | | | | | |
